# Kras mutation correlating with circulating regulatory T cells predicts the prognosis of advanced pancreatic cancer patients

**DOI:** 10.1002/cam4.2895

**Published:** 2020-02-03

**Authors:** He Cheng, Guopei Luo, Kaizhou Jin, Zhiyao Fan, Qiuyi Huang, Yitao Gong, Jin Xu, Xianjun Yu, Chen Liu

**Affiliations:** ^1^ Department of Pancreatic Surgery Fudan University Shanghai Cancer Center Shanghai China; ^2^ Department of Oncology Shanghai Medical College Fudan University Shanghai China; ^3^ Shanghai Pancreatic Cancer Institute Shanghai China

**Keywords:** Kras mutation, pancreatic cancer, prognosis, Tregs

## Abstract

**Purpose:**

Kras mutation and abnormal immune status are associated with pancreatic cancer development and progression. In this study, we evaluated the Kras mutation status in circulating tumor DNA and circulating T cell subsets in a cohort of advanced pancreatic cancer patients.

**Methods:**

Samples were retrospectively obtained from a series of 210 pathological advanced pancreatic cancer patients between 2012 and 2014. The Kras mutation status was detected in cell‐free circulating tumor DNA (ctDNA) by ddPCR and circulating T cell subsets were analyzed by flow cytometry.

**Results:**

Univariate analysis found that tumor node metastasis (TNM) stage, chemotherapy, circulating regulatory T cells, CA19‐9 levels, CA125 levels, and Kras^G12D^ and Kras^G12V^ mutations were significantly related to overall survival in advanced pancreatic cancer patients. Multivariate analysis identified that TNM stage (*P* = .03, HR:1.422), Tregs (*P* = .004, HR:1.522), CA19‐9 levels (*P* = .009, HR:1.488), Kras^G12D^ mutation (*P* = .044, HR:1.353), and Kras^G12V^ mutation (*P* = .001, HR:1.667) were independent prognostic markers. Furthermore, we found that Kras^G12V^ mutation in ctDNA was correlated with high circulating proportion of Tregs, and patients with both Kras^G12V^ mutation and high levels of Tregs were associated with extremely poor survival in advanced pancreatic cancer.

**Conclusion:**

Kras^G12V^ mutation was associated with high circulating regulatory T cell levels, and both of them predicted worse prognosis in advanced pancreatic cancer patients.

## Graphical abstract

KrasG12V mutation in ctDNA was correlated with high circulating proportion of Tregs. Patients with both KrasG12V mutation and high levels of Tregs were associated with extremely poor survival in advanced pancreatic cancer.

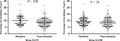

## 1 INTRODUCTION

Pancreatic cancer is one of the most lethal cancer with an extremely poor prognosis. It was supposed to be the second leading cause of cancer‐related deaths in the USA by the year 2020.^1^ About 80%‐85% of patients are diagnosed at advanced stage because of lacking specific symptoms, and lose the opportunity for radical surgery.^2^ Chemotherapy is the preferred option for these patients and there has been great progress in recent years.^3^, ^4^ However, 5‐year survival rate in advanced pancreatic cancer is still less than 5%. As it is invasive and uneasy to obtain enough tumor tissues in advanced pancreatic cancer, CA19‐9 is the most used noninvasive prognostic markers in these patients but with several limitations.^5^ It is necessary to identify other circulating prognostic biomarkers.

Cell‐free circulating tumor DNA (ctDNA), also known as liquid biopsy, is a noninvasive biomarker in various cancer.^6^ It was reported that specific gene mutations in ctDNA can be used as diagnostic and prognostic markers in pancreatic cancer.^7^ Kras is the most frequently reported oncogenic mutation in ctDNA of pancreatic cancer with the rate ranging from 65% to 85%.^8^ Several studies identified that Kras mutation in ctDNA plays a prognostic role in pancreatic cancer.^9^ However, Kras‐related target therapy or immune treatment almost failed to improve survival in clinical trials.^10^, ^11^

Immune disorder frequently occurred in pancreatic cancer and associated with the tumor progression and development. Abnormal distribution of T cell subsets such as high level of regulatory T cells (Tregs) and low level of cytotoxic T cells contributed to immunosuppressive environment in pancreatic cancer, and led to the escape of tumor cells from immune surveillance.^12^ It was reported that mutated Kras is associated with T cell differentiation and function in colorectal and lung cancers.^13^ Our previous study also found that Kras mutation correlates with Tregs infiltration in resectable pancreatic cancer tissues.^14^ However, the possible association of Kras mutation and T cell subsets distribution in circulating peripheral blood of pancreatic cancer has not been elaborated to date. Therefore, in this study, we focused on the potential correlation between the Kras mutation in ctDNA and circulating T cell subsets in a cohort of Chinese patients with advanced pancreatic cancer.

## 2 MATERIAL AND METHODS

### 2.1 Study population

This study included 210 advanced pancreatic cancer patients with pathologically confirmed adenocarcinoma in our center from 2012 to 2014. All the patients did not receive any anticancer treatments before the first hospitalization in our center. Tumor node metastasis (TNM) stage was defined by AJCC TNM staging of pancreatic cancer 2018, and patients with stage III and IV were included. Overall survival (OS) was measured by the date of diagnosis to the time of death, and the clinical parameters were obtained from electronic records. The final date of follow‐up was January 2019. Written informed consent was obtained from each patient. This study was approved by the Clinical Research Ethic Committee of Shanghai Cancer Center.

### 2.2 T cell subsets detected by flow cytometry

Peripheral blood samples were collected in heparinized tubes at admission, and processed for flow cytometry within 2 hours. To identify different T cell subsets, anti‐CD3, anti‐CD4, anti‐CD8, anti‐CD25, and anti‐CD127 from BD Bioscience were used. A minimum of 10,000 events gated on the population of interest were analyzed. The experimental steps for flow cytometry to identify different T cell subsets in peripheral blood sample have been described in detail previously.^15^

### 2.3 CTDNA mutation detected using droplet digital PCR (DDPCR)

Circulating DNA was isolated and collected from about 5 mL of plasma according to the QIAamp Circulating Acid Kit (Qiagen), and then processed to droplet digital PCR to detect the Kras mutation levels of circulating tumor DNA. Primers and probes for detection of Kras^G12V^ and Kras^G12D^ mutation were acquired following the experimental protocol (Bio‐Rad Laboratories). Kras‐G12V‐F (Forward primer): TGCTGAAAATGACTGAATATAAACTTGTG, Kras‐G12V‐R (Reverse primer): AGCTGTATCGTCAAGGCACTCTT and Kras‐G12V‐P (Probe): TTGGAGCTGTTGGC; Kras‐G12D‐F: TGCTGAAAATGACTGAATATAAACTTGTG, Kras‐G12V‐D: AGCTGTATCGTCAAGGCACTCTT and Kras‐G12D‐P: TGGAGCTGATGGCGT. Detailed steps for ddPCR were previously described.^8^ For the threshold of ddPCR determination, positive result was identified as PCR monodispersed droplets had a fluorescence signal, while none fluorescence signal represented none mutation (Figure S1 and S2).

### 2.4 Statistical analysis

The statistical analyses were conducted using SPSS version 19.0 software. Kaplan‐Meier method was used to plot the survival curve. The independent prognostic factors were identified through univariate and multivariate analyses using the Cox proportional hazard regression model. Continuous variable data between two groups were compared by the student's t test. Significant difference was defined as a *P*‐value < .05.

## 3 RESULTS

### 3.1 Patient characteristics

We retrospectively collected data from 210 advanced pancreatic cancer patients including 71 locally advanced and 139 metastatic cases. The basic features of these patients are listed in Table 1. The median age of this group patients was 63 years old (range from 33 to 79 years). At the time of last follow‐up, all the patients died. Among the 210 patients, 178 (84.8%) patients received gemcitabine‐based or 5‐FU‐based chemotherapy, and other 32 (15.2%) patients accepted only best supportive care. In addition, we also detected the Kras mutation status in ctDNA and circulating T cell subsets in this group patients; the Kras^G12V^ mutation was detected in 61 (29%) cases and Kras^G12D^ mutation in 93 (44.3%) cases. The mean values of CD3 + CD4+ T cells, CD3+ CD8+ T cells, and Tregs were 38.9%±9.0%, 22.7%±9.2%, and 9.1%±3.3%, respectively.

**Table 1:** Clinicopathological parameters of patients with advanced pancreatic cancer (n = 210)

Parameter	Category	No	%
Age	<65	139	66.2%
	≥65	71	33.8%
Gender	Male	132	62.9%
	Female	78	37.1%
Stage	III	71	33.8%
	IV	139	66.2%
Chemotherapy	Yes	178	84.8%
	No	32	15.2%
CA19‐9 level	<1000 U/mL	130	61.9%
	≥1000 U/mL	80	38.1%
CA125 level	<35 U/mL	88	41.9%
	≥35 U/mL	122	58.1%
Kras G12V	Mutation	61	29%
	None G12V mutation	149	71%
Kras G12D	Mutation	93	44.3%
	None G12D mutation	117	55.7%

### 3.2 The prognostic role of KRAS mutation and circulating T cell subsets in patients with advanced pancreatic cancer via univariate and multivariate analyses

**Figure 1:**
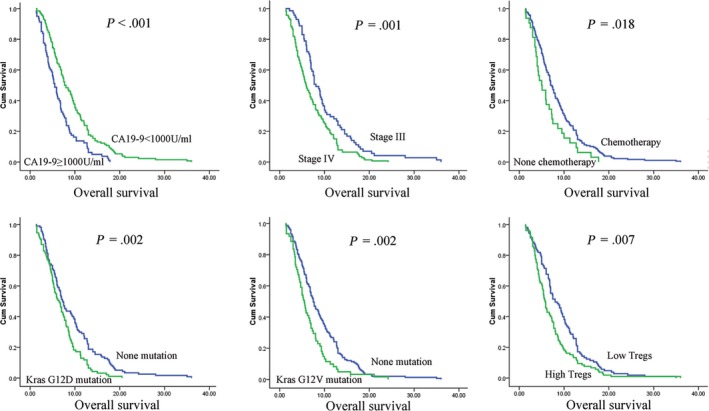
Kaplan‐Meier analyses of the overall survival difference in patients with advanced pancreatic cancer. Groups were compared by univariate analysis

The cutoff for CA19‐9 and CA125 were 1000 U/mL and 35 U/mL according to our previous studies.^16^, ^17^ We chose the median value of CD3+ CD4+ T cells (38.99%), CD3+ CD8+ T cells (21.06%), and Tregs (8.66%) as cutoff, respectively. The association between various clinicopathological factors and OS is shown in Table 2. Overall survival curves are presented by Kaplan‐Meier analysis in Figure 1. Univariate analysis revealed that TNM stage, chemotherapy, Tregs, CA19‐9 levels, CA125 levels, and Kras^G12V^ and Kras^G12D^ mutations were significantly associated with OS, while age, gender, CD3+ CD4+ T cells, and CD3+ CD8+ T cells have no sense for prognosis. Furthermore, multivariate analysis identified stage IV (*P* = .03), high proportion of Tregs (*P* = .004), CA19‐9 ≥ 1000U/ml (*P* = .009), Kras^G12V^ mutation (*P* = .001), and Kras^G12D^ mutation (*P* = .044) as independent poor prognostic factors for OS in these advanced pancreatic cancer cases.

**Table 2:** Univariate and multivariate analyses of clinicopathological parameters for the prediction of overall survival in patients with advanced pancreatic cancer (n = 210)

Parameters	Univariate analyses		Multivariate analyses	
	*P*	HR (95%CI)	*P*	HR (95%CI)
Age (years): <65 vs ≥65	.145	—	—	—
Gender: Male vs Female	.766	—	—	—
TNM stage: IV vs III	.001	1.626 (1.212‐2.179)	.03	1.422 (1.034‐1.957)
Chemotherapy: Yes vs No	.018	0.632 (0.432‐0.924)	.066	0.698 (0.476‐1.025)
Tregs: High vs Low (Median：8.66%)	.007	1.458 (1.109‐1.912)	.004	1.522 (1.143‐2.028)
CD3+ CD4+ T cells: High vs Low (Median：38.99%)	.211	1.189 (0.906‐1.56)	—	—
CD3+ CD8+ T cells: High vs Low (Median：21.06%)	.494	0.909 (0.69‐1.196)	—	—
Kras G12V	.002	1.616 (1.192‐2.183)	.001	1.667 (1.217‐2.028)
Mutation vs None				
Kras G12D	.002	1.577 (1.188‐2.092)	.044	1.353 (1.009‐1.815)
Mutation vs None				
CA19‐9 level (U/mL)	<.001	1.822 (1.367‐2.429)	.009	1.488 (1.103‐2.008)
≥1000 vs <1000				
CA125 level (U/mL)	<.001	0.576 (0.434‐0.764)	.055	0.747 (0.555‐1.007)
<35 vs ≥35				

Abbreviation: 95%CI, 95% confidence interval; HR: hazard ratio.

### 3.3 The status of KRAS mutation correlates with circulating regulatory T cells to further stratify OS in patients with advanced pancreatic cancer patients

**Figure 2:**
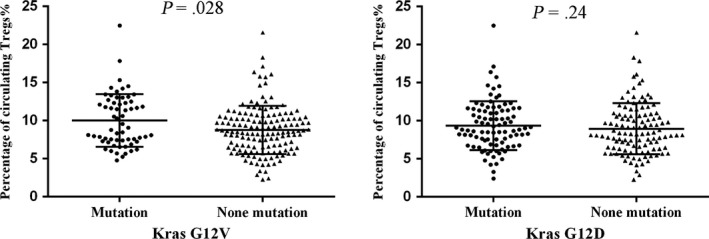
Kras^G12V^ mutation was associated with a high proportion of Tregs, while Kras^G12D^ mutation was not

**Figure 3:**
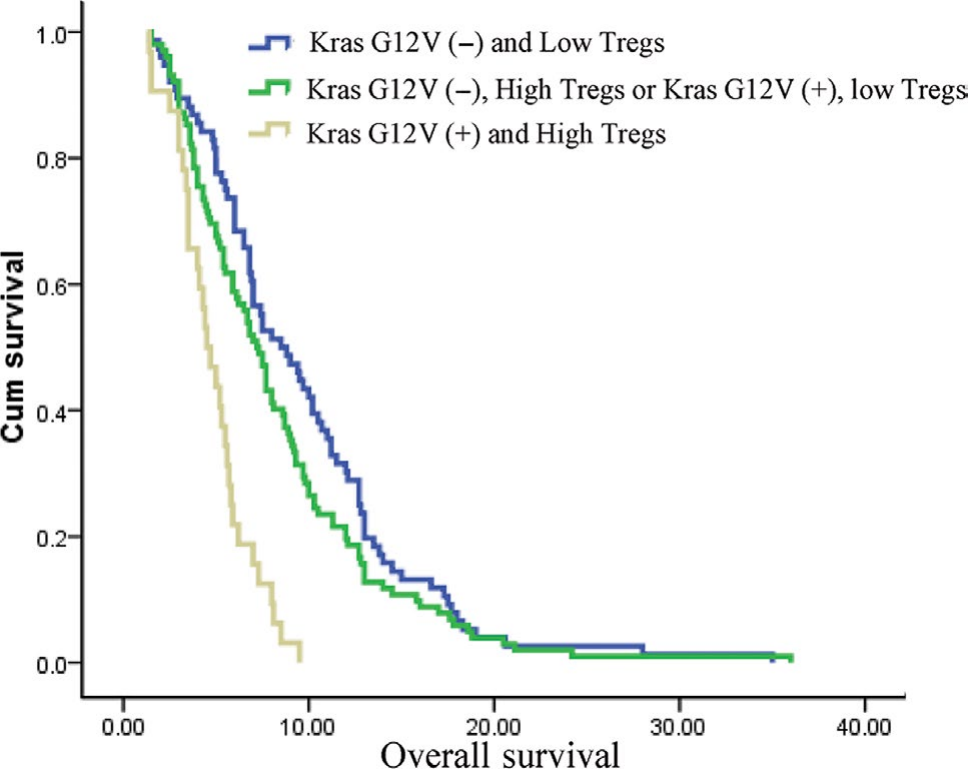
Combination of Kras^G12V^ mutation and regulatory T cells further stratify prognosis in advanced pancreatic cancer patients

It was reported that Kras mutation was associated with Tregs infiltration in various tumor tissues.^13^, ^14^, ^18^ Therefore, we analyzed the potential correlation between Kras mutation status and Tregs distribution in peripheral blood samples in advanced pancreatic cancer. Interestingly, we found that Kras^G12V^ mutation was notably associated with high levels of Tregs (*P* = .028), while Kras^G12D^ had no relationship with Tregs (Figure 2). As both Kras^G12V^ mutation and Tregs were independent prognostic factors in this study, patients were divided into three groups: 1. Kras^G12V^ mutation (+) and high Tregs; 2. Kras^G12V^ mutation (+), low Tregs or Kras^G12V^ mutation (−), high Tregs; 3. Kras^G12V^ mutation (−) and low Tregs. Kaplan‐Meier analysis with a log‐rank test found that patients with both Kras^G12V^ mutation and high Tregs (n = 32) had the worst survival with a median OS of 4.5 m (95%CI: 3.53‐5.47 m), whereas those with none Kras^G12V^ mutation and low Tregs (n = 76) had a median OS of 8.5 m (95%CI: 6.26‐10.73 m; *P* < .001), predicting a better prognosis (Figure 3 and Table 3).

**Table 3:** The overall survival stratified by combination of Kras^G12V^ mutation and Tregs

Group	Number	Median OS (mon)	95% Confidence Interval
1. KrasG12V (−) and Low Tregs	76	8.5	6.26‐10.74
2. KrasG12V (−), High Tregs or KrasG12V (+), Low Tregs	102	7.2	6.29‐8.11
3. KrasG12V (+) and High Tregs	32	4.5	3.53‐5.47

Note

*P* < .001.

## 4 DISCUSSION

The genetic landscape of pancreatic cancer is notable for activating Kras mutation and inactivation of smad4, TP53, and CDKN2A. Among these four driver genes, Kras is the most frequent mutated gene, and runs through the initiation, progression, and metastasis of pancreatic cancer.^19^ Scientists had already been aware of the importance of Kras mutation in pancreatic cancer, and inhibition of Kras activity in mice model of pancreatic cancer induced tumor regression.^20^ However, almost all treatments against Kras failed to improve prognosis in clinical trials. It was reported that Kras mutation activates several key pathways to allow tumor cells growth and metastasis.^11^ The prognostic role of Kras mutation in pancreatic cancer is still controversy and inconsistent.^21^ It was reported that Kras mutation detected in pancreatic cancer tissues associated with worse disease‐free survival and OS compared with Kras wild‐type tumors. In addition, subtype analysis revealed that patients with Kras^G12D^ mutation had an extremely poor prognosis with a median OS of 15.3 months in resectable pancreatic cancer, while other studies showed different results.^22^, ^23^ Change of Kras mutation in ctDNA could also be used to monitor treatment response in metastatic pancreatic cancer and Kras mutation detected in ctDNA after surgery is associated with early recurrence and metastasis.^8^, ^24^ Two patterns (G12V and G12D) of Kras mutation account for about 90% of all mutations in pancreatic cancer and both mutation rates range from 30% to 50%. Therefore, in this study, we detected these two mutation sites in ctDNA of advanced pancreatic cancer, and found that both Kras^G12D^ mutation and Kras^G12V^ mutation were associated with poor prognosis, which was consistent with other studies.^25^

Pancreatic cancer is characteristically surrounded by abundant stroma, which caused a hypoxia status and abnormal immune environment.^26^ T cell subset abnormal distribution and dysfunction are important features of immunosuppresive status in pancreatic cancer.^15^ Tregs is a classic immune‐suppressive T cell subset, which secretes various cytokines to inhibit CD8+ T cell function and allows tumor cells escape from immune surveillance.^27^ It was reported that high Tregs infiltration in tumor tissues was associated with poor OS.^12^ In this study, we found that high proportion of Tregs in peripheral blood was an independent negative prognostic factor for advanced pancreatic cancer patients.

Increasing evidences revealed that there is a crosstalk between Kras mutation and T cell immune disorder in Kras mutation tumors.^13^, ^28^ Pancreatic cancer cells with oncogenic Kras mutation secrete various important molecules to affect components of the stroma, such as innate and adaptive immune cells.^29^, ^30^ These cells in turn promote and maintain tumor growth and metastasis. Several studies identified that Kras^G12D^ or Kras^G12V^ mutation contributes to T cell differentiation in colorectal and lung cancer cells.^13^, ^31^ Our previous studies also found that Kras^G12D^ mutation is associated with high Tregs infiltration in resectable pancreatic cancer tissues.^14^ However, the potential correlation of Kras mutation and T cell subsets is still unclear in advanced pancreatic cancer. Endoscopic ultrasound‐guide fine‐needle aspiration (EUS‐FNA) is an invasive approach and often obtain insufficient tissues for infiltrating immune cell and Kras mutation detection, and therefore, we identified Kras mutation status in ctDNA and also detected T cell subsets proportion in peripheral blood samples. We found that Kras^G12V^ mutation, not Kras^G12D^, was associated with high proportion of Tregs. In addition, Kras^G12V^ mutation combined with a high proportion of Tregs correlated strongly with poor survival.

Palliative chemotherapy is the main and standard treatment for advanced pancreatic cancer, but the outcomes are diverse from suboptimal. Patients with adverse prognostic factors, such as Kras mutation and high Tregs, might benefit from more aggressive multiagent scheme. Moreover, understanding the detailed molecular events of patients with high‐risk negative prognostic factors in advanced pancreatic cancer may help guide the treatment strategy and improve OS.

There are several limitations in this study. Firstly, this is a retrospective study with relatively low evidence grade and lack of continuous samples after chemotherapy for monitoring treatment responses. Secondly, as tumor tissues or cells obtained by EUS‐FNA are few and mostly used for diagnosis, it was uneasy to detect the T cell infiltration in pancreatic cancer tissues. Therefore, we were unable to detect the correlation of Kras mutation and T cell infiltration in advanced pancreatic cancer. At last, the potential mechanism underlying this correlation is not elaborated in this clinical study.

## 5 CONCLUSION

In summary, this study identified potential circulating biomarkers to predict prognosis in advanced pancreatic cancer. We found that Kras^G12V^ mutation in ctDNA was correlated with suppressive immune status marked with high proportion of Tregs in peripheral blood for the first time. Combining these two factors could further stratify advanced pancreatic cancer into different prognostic subgroups. Further studies should demonstrate the detailed mechanism about the relationship between Kras mutation and immune disorder.
