# Supplementary figures and images for: Kras mutation correlating with circulating regulatory T cells predicts the prognosis of advanced pancreatic cancer patients
Source: Cancer Med. 2020 Feb 3;9(6):2153–9. doi: 10.1002/cam4.2895 (PMC7064028; doi:10.1002/cam4.2895)

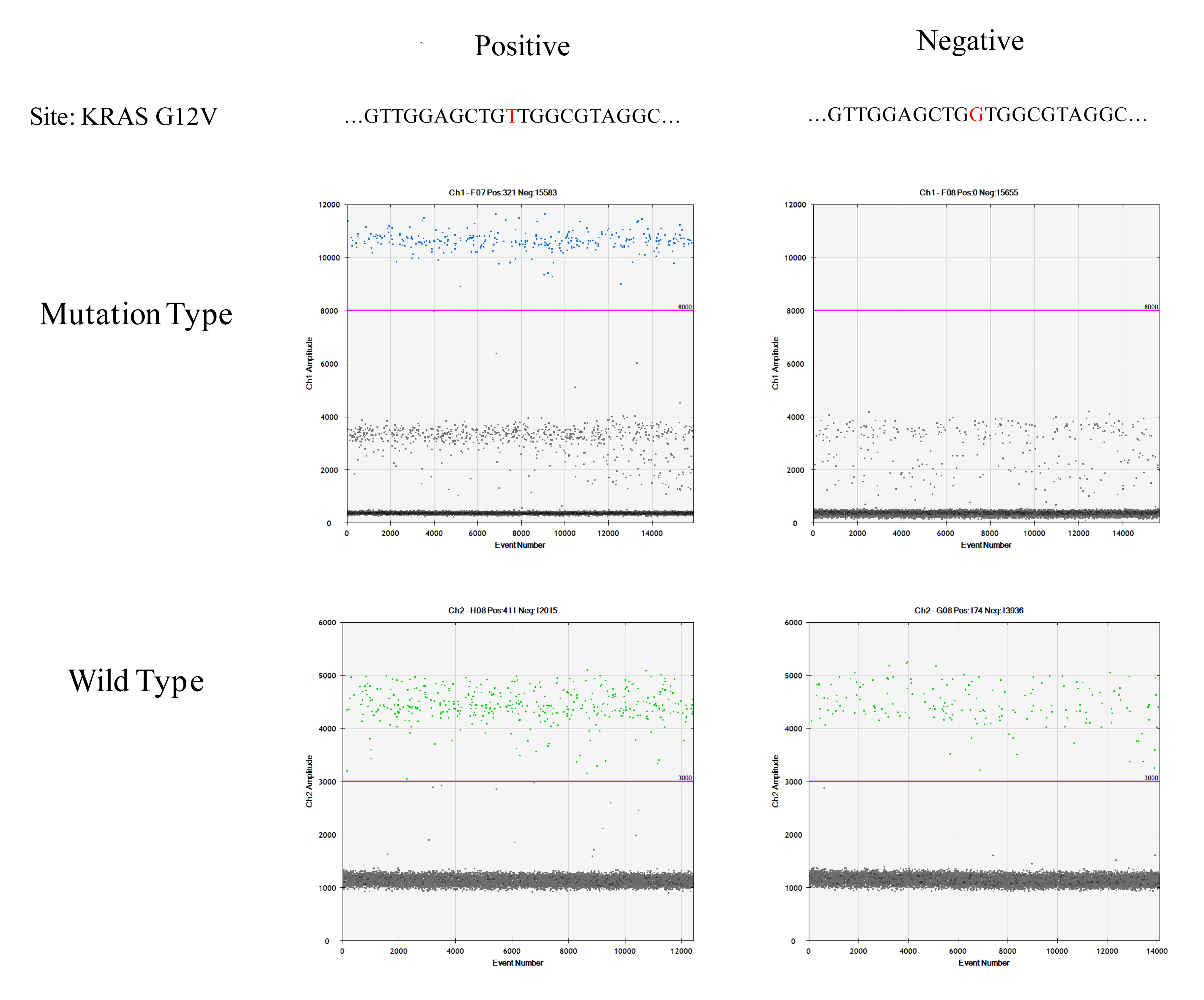

Supplement: Supplementary file 1 [file CAM4-9-2153-s001.tif]

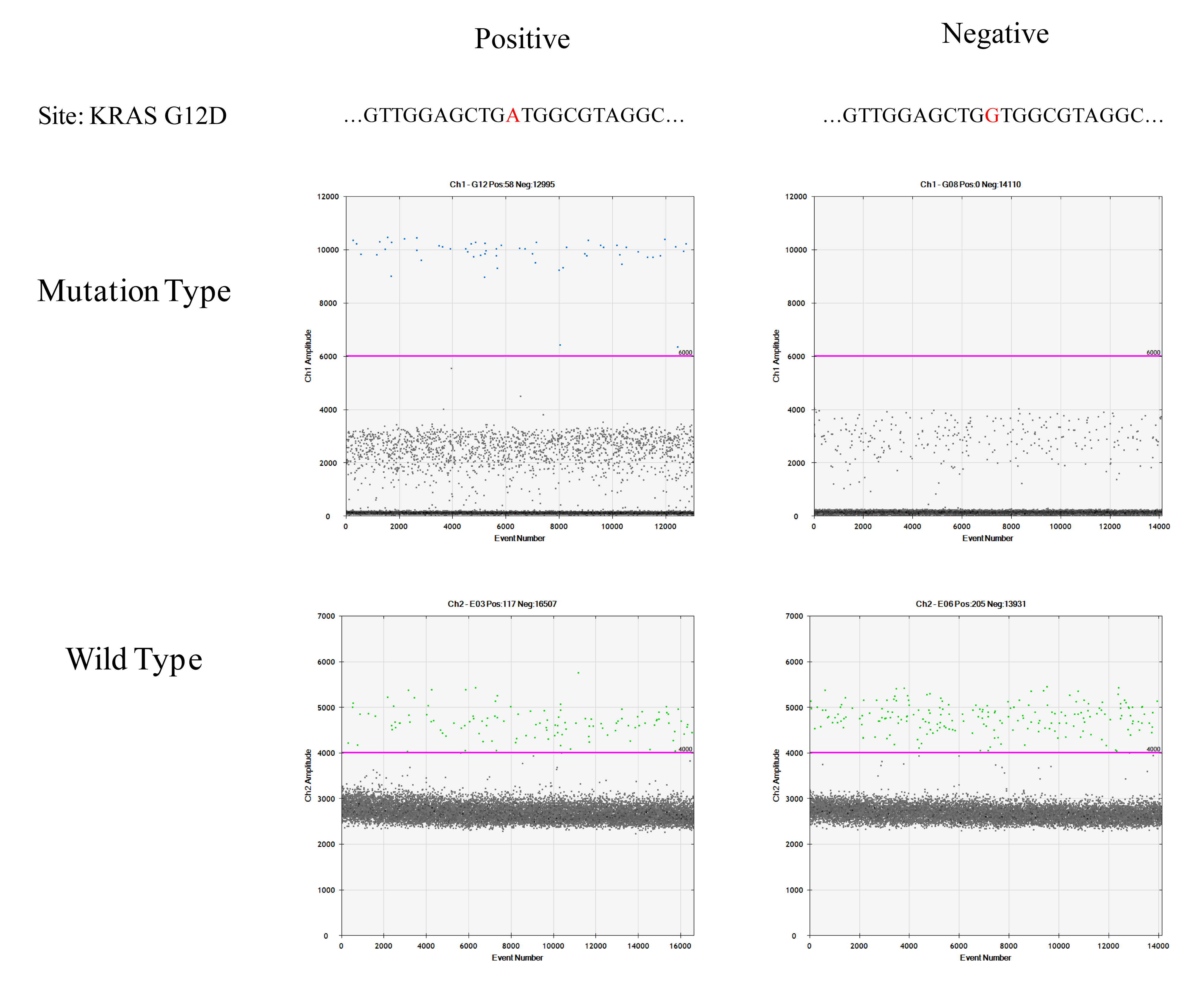

Supplement: Supplementary file 2 [file CAM4-9-2153-s002.tif]
