# Supplementary material for: Kras mutation correlating with circulating regulatory T cells predicts the prognosis of advanced pancreatic cancer patients
Source: Cancer Med. 2020 Feb 3;9(6):2153–9. doi: 10.1002/cam4.2895 (PMC7064028; doi:10.1002/cam4.2895)
Supplement: Supplementary file 3 [file CAM4-9-2153-s003.docx]

**Supplementary Figure Legends**

Supplementary Fig. S1: Typical examples of Kras^G12V^ mutation

Supplementary Fig. S2: Typical examples of Kras^G12D^ mutation
